# Supplementary material for: Unveiling the Stereoselectivity and Regioselectivity of the [3+2] Cycloaddition Reaction between N-methyl-C-4-methylphenyl-nitrone and 2-Propynamide from a MEDT Perspective
Source: Int J Mol Sci. 2023 May 22;24(10):9102. doi: 10.3390/ijms24109102 (PMC10218864; doi:10.3390/ijms24109102)
Supplement: Supplementary file 1 [file ijms-24-09102-s001.zip › ijms-2398226-supplementary.pdf]

Unveiling the Stereoselectivity and Regioselectivity of the [3+2] Cycloaddition Reaction between N-methyl-C-4-methylphenyl-nitrone and 2-propynamide with a MEDT Perspective

Sabir A. Mohammed Salih[1], Huda A. Basheer[1], Jesus Vicente de Julian Ortiz[2],

Haydar A. Mohammad-Salim[1,2*] .

[1]Faculty of Science, Department of Chemistry, University of Zakho, Duhok 42001, Iraq.

[2]Molecular Topology and Drug Design Research Unit, Department of Physical Chemistry, Pharmacy Faculty, University of Valencia, 46100 Valencia, Spain

The equilibrium geometries optimized at DFT B3LYP/6-311++G (d, p) level of all reactants, products and transition states used in this research, as well as, the summary of calculations: are listed below:

Optimized Geometries for reactants, products and TSs:

Reactant **1**

Atom	Coordinates (Angstroms)		
	X	Y	Z
C	-2.61835400	-0.09877900	0.00931000
C	-1.70053900	-1.15720600	0.01286200
C	-0.32523200	-0.94021700	0.00787900
C	0.18383500	0.37557400	0.00203700
C	-0.74029300	1.44273700	0.00330100
C	-2.10862400	1.20865200	0.00803400
H	-2.07067400	-2.18026200	0.02100000
H	0.37039000	-1.76814900	0.01224400
H	-0.37233000	2.46677200	0.00471500

The geometry of the reactant **1**

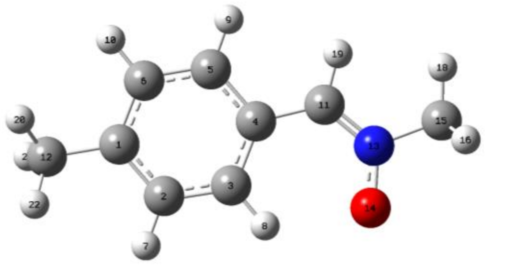

H	-2.79570000	2.05209400	0.01222500
C	1.59134000	0.71605700	-0.00049000
C	-4.10650300	-0.35245000	-0.01814200
N	2.60641300	-0.12621700	-0.00292200
O	2.53426200	-1.40130400	-0.00285900
C	3.99142200	0.39437600	-0.00632800
H	4.48969700	-0.00128400	0.88106800
H	4.48514800	-0.00072300	-0.89651700
H	4.01129600	1.48553400	-0.00602800
H	1.86412200	1.76435000	-0.00148900
H	-4.65577400	0.41575100	0.53739100
H	-4.49249500	-0.34547600	-1.04674600

Electronic Energy (EE) = -479.63096 Hartree

Zero-point Energy Correction = 0.181827 Hartree

Thermal Correction to Energy = 0.19245 Hartree

Thermal Correction to Enthalpy = 0.193395 Hartree

Thermal Correction to Free Energy = 0.144683 Hartree

EE + Zero-point Energy = -479.44913 Hartree

EE + Thermal Energy Correction = -479.43851 Hartree

EE + Thermal Enthalpy Correction = -479.43756 Hartree

EE + Thermal Free Energy Correction = -479.48627 Hartree

E (Thermal) = 120.764 kcal/mol

Heat Capacity (Cv) = 39.408 cal/mol-kelvin

Entropy (S) = 102.523 cal/mol-kelvin

Reactant **2**

Atom	Coordinates (Angstroms)		
	X	Y	Z
C	2.17665700	0.06959100	-0.00001900
C	0.97094900	-0.00410500	-0.00001300
C	-0.48501300	-0.14673800	0.00000700
N	-1.15451000	1.04367300	-0.00000800
O	-1.03747900	-1.23620400	0.000 01900
H	3.24281500	0.11674700	-0.00002200
H	-2.16459800	1.02510200	0.00004100
H	-0.67236800	1.92958700	0.00003400

The geometry of the reactant **2**

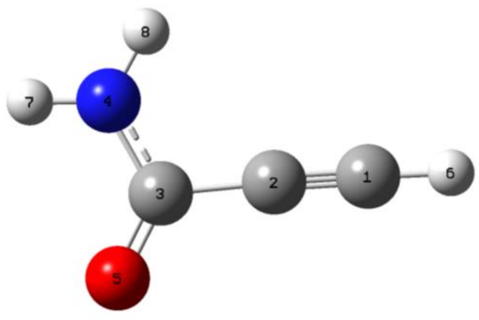

Electronic Energy (EE) = -246.11532 Hartree

Zero-point Energy Correction = 0.054762 Hartree

Thermal Correction to Energy = 0.060229 Hartree

Thermal Correction to Enthalpy = 0.061173 Hartree

Thermal Correction to Free Energy = 0.026599 Hartree

EE + Zero-point Energy = -246.06056 Hartree

EE + Thermal Energy Correction = -246.05509 Hartree

EE + Thermal Enthalpy Correction = -246.05415 Hartree

EE + Thermal Free Energy Correction = -246.08872 Hartree

E (Thermal) = 37.794 kcal/mol

Heat Capacity (Cv) = 18.347 cal/mol-kelvin

Entropy (S) = 72.766 cal/mol-kelvin

Product **3**

Atom	Coordinates (Angstroms)		
	X	Y	Z
C	3.85500100	-0.52302800	-0.21375800
C	2.81452300	-0.81311300	-1.11149300
C	1.50030300	-0.45259200	-0.83356800
C	1.18235900	0.215413 00	0.35702400
C	2.21207000	0.51209200	1.25152000
C	3.53051500	0.14471100	0.97066100
H	3.04110800	-1.33037000	-2.04168500
H	0.70870300	-0.67613700	-1.54232600
H	1.98545600	1.03381400	2.17929600
H	4.31656900	0.38520100	1.68292800

The geometry of the product **3**

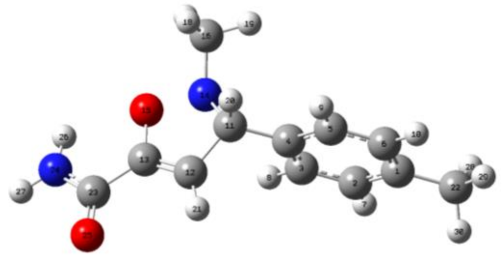

C	-0.25277400	0.59211300	0.68183400
C	-1.22409000	-0.55390300	0.83920900
C	-2.34810500	-0.24147900	0.19160400
N	-0.86709100	1.34126700	-0.45598300
O	-2.30277800	0.96546100	-0.45631500
C	-0.83262200	2.78575400	-0.29619800
H	-1.34592000	3.25572900	-1.13889900
H	-1.28761100	3.12624900	0.64769100
H	0.22002300	3.08467500	-0.31809300
H	-0.24599200	1.20369400	1.60764500
H	-1.05490500	-1.44757400	1.42110200
C	5.27870500	-0.91853400	-0.52957500
C	-3.60220500	-1.04246900	0.07214200
N	-4.53800900	-0.49406100	-0.75421500
O	-3.75287600	-2.09246300	0.68187100
H	-4.32242800	0.31510600	-1.31817200
H	-5.37958500	-1.02095700	-0.93709900
H	5.64173900	-0.41729000	-1.43577100
H	5.95734600	-0.65832400	0.28880100
H	5.36436400	-1.99803100	-0.70487600

Electronic Energy (EE) = -725.80209 Hartree

Zero-point Energy Correction = 0.241943 Hartree

Thermal Correction to Energy = 0.257455 Hartree

Thermal Correction to Enthalpy = 0.258399 Hartree

Thermal Correction to Free Energy = 0.197208 Hartree

EE + Zero-point Energy = -725.56015 Hartree

EE + Thermal Energy Correction = -725.54464 Hartree

EE + Thermal Enthalpy Correction = -725.54369 Hartree

EE + Thermal Free Energy Correction = -725.60488 Hartree

E (Thermal) = 161.555 kcal/mol

Heat Capacity (Cv) = 58.11 cal/mol-kelvin

Entropy (S) = 128.787 cal/mol-kelvin

Product **4**

Atom	Coordinates (Angstroms)		
	X	Y	Z
C	3.05611840	-0.21524648	-0.14610954
C	2.10807940	-0.22284348	-1.18038554
C	0.76674540	-0.00684248	-0.89326854
C	0.34243740	0.22914452	0.42035446
C	1.29716740	0.23456752	1.44335246
C	2.64282940	0.01218352	1.17313346
H	2.42887640	-0.40435248	-2.19880454
H	0.03890440	-0.03419148	-1.69564154
H	0.98389040	0.41403452	2.46714146
H	3.37326840	0.01278252	1.97278946
C	-1.12439760	0.50407252	0.75628946
C	-2.11453160	-0.34235448	-0.02736154
C	-2.72728560	0.46254152	-0.90809554
N	-1.63657160	1.87996352	0.43961646
O	-2.38331160	1.75436552	-0.82889954
C	-2.36560060	-1.79251048	0.05451046
O	-3.24336260	-2.34168148	-0.59732754
N	-1.57920360	-2.49067048	0.94202046
C	-0.67972760	2.95884552	0.25102646
H	-0.70083560	-2.11791648	1.26671846
H	-1.66986360	-3.49547348	0.91592046
H	-1.25058960	0.38860752	1.83775846
H	0.05103140	2.77653652	-0.54588854

The geometry of the product **4**

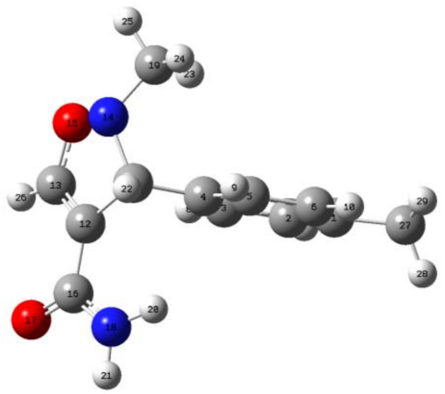

Electronic Energy (EE) = -725.80069 Hartree

Zero-point Energy Correction = 0.242931 Hartree

Thermal Correction to Energy = 0.25814 Hartree

Thermal Correction to Enthalpy = 0.259085 Hartree

Thermal Correction to Free Energy = 0.199381 Hartree

EE + Zero-point Energy = -725.55776 Hartree

EE + Thermal Energy Correction = -725.54255 Hartree

EE + Thermal Enthalpy Correction = -725.5416 Hartree

EE + Thermal Free Energy Correction = -725.60131 Hartree

E (Thermal) = 161.986 kcal/mol

Heat Capacity (Cv) = 57.632 cal/mol-kelvin

Entropy (S) = 125.656 cal/mol-kelvin

H	-0.15274360	3.09695252	1.19727146
H	-1.23734060	3.86996652	0.03304346
H	-3.44895260	0.20982252	-1.67153454
C	4.53271457	-0.37047268	-0.06223462
H	4.88936899	-1.37928269	-0.06223462
H	4.88938741	0.13392551	0.81141689
H	4.88938741	0.13392551	-0.93588612

Product **5**

Atom	Coordinates (Angstroms)		
	X	Y	Z
C	-3.41209731	-0.48175573	-0.08328083
C	-3.26356631	0.64648027	0.73447017
C	-1.98974631	1.09702927	1.05967417
C	-0.84781531	0.44121027	0.58838217
C	-1.00804131	-0.69093673	-0.21949383
C	-2.27420831	-1.14981673	-0.55903383
H	-4.14166331	1.15743227	1.109 79817
H	-1.88186031	1.96981327	1.69566117
H	-0.13511931	-1.22491473	-0.57554883
H	-2.39059331	-2.02644373	-1.18437483
C	0.53562069	0.98433427	0.94798817
C	1.54636369	-0.09373973	1.25068217
C	2.40814969	-0.16111873	0.23637717
N	1.26968069	1.74806627	-0.12061583
O	2.19282669	0.77347427	-0.73865883

The geometry of the product **5**

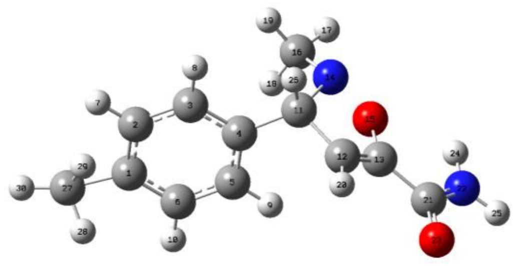

C	0.49750269	2.33027527	-1.20648883
H	1.18557869	2.84768027	-1.87591283
H	-0.09177831	1.60234027	-1.77667683
H	-0.17459131	3.07070227	-0.76747383
H	1.53102069	-0.74874873	2.10632217
C	3.55004469	-1.10723073	0.04776317
N	4.32822369	-0.83855573	-1.03551283
O	3.73733369	-2.03648373	0.81319217
H	4.17278769	-0.01733873	-1.59835683
H	5.13860869	-1.41483473	-1.19906383
H	0.41571069	1.68487727	1.78008417
C	-4.84684530	-0.82830382	-0.26457902
H	-4.49019088	-1.83711382	-0.26457902
H	-4.49017246	-0.32390563	0.60907248
H	-5.91684530	-0.82829063	-0.26457902

Electronic Energy (EE) = -725.79899 Hartree

Zero-point Energy Correction = 0.242276 Hartree

Thermal Correction to Energy = 0.256786 Hartree

Thermal Correction to Enthalpy = 0.257731 Hartree

Thermal Correction to Free Energy = 0.200023 Hartree

EE + Zero-point Energy = -725.55671 Hartree

EE + Thermal Energy Correction = -725.5422 Hartree

EE + Thermal Enthalpy Correction = -725.54126 Hartree

EE + Thermal Free Energy Correction = -725.59896 Hartree

E (Thermal) = 161.136 kcal/mol

Heat Capacity (Cv) = 56.049 cal/mol-kelvin

Entropy (S) = 121.455 cal/mol-kelvin

Product **6**

Atom	Coordinates (Angstroms)		
	X	Y	Z
C	3.07842592	-0.01049829	-0.12228563
C	2.56483392	-0.06257029	1.17877737
C	1.19692292	-0.23587429	1.37266737
C	0.32716492	-0.35319029	0.28537237
C	0.85019392	-0.30578029	-1.01230163
C	2.21078392	-0.13622429	-1.22004563
H	3.23394692	0.02574871	2.02572337

The geometry of the product **6**

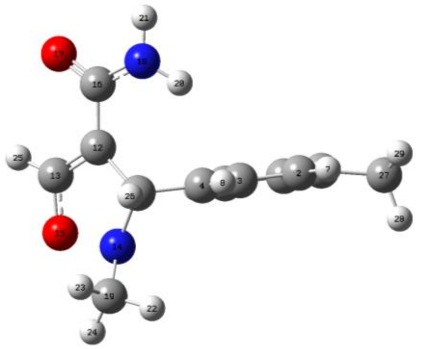

H	0.80392392	-0.28423329	2.38317537
H	0.18208092	-0.41213029	-1.85808263
H	2.61231192	-0.09994329	-2. 22524663
C	-1.16709808	-0.52799829	0.51379337
C	-2.08396508	0.52839571	-0.08951663
C	-3.06388708	-0.11970229	-0.73578763
N	-1.63178208	-1.76409129	-0.18186263
O	-2.95898308	-1.45341529	-0.75043063
C	-2.09253808	1.99362171	0.08970037
O	-2.99183808	2.69432771	-0.35555563
N	-1.05975508	2.52516971	0.82455237
C	-1.81826608	-2.91464629	0.68913237
H	-0.19620608	2.02095771	0.95306537
H	-1.01422608	3.53310571	0.85127237
H	-0.83334208	-3.19654329	1.06781137
H	-2.48925508	-2.70634829	1.53443637
H	-2.21284208	-3.74475929	0.10350237
H	-3.92890008	0.29064371	-1.23680263
H	-1.33984808	-0.59701429	1.60248737
C	4.53515118	-0.11223968	-0.40347349
H	4.89180561	-1.12104968	-0.40347349
H	4.89182402	0.39215851	0.47017801
H	4.89182402	0.39215851	-1.27712500

Electronic Energy (EE) = -725.80431 Hartree

Zero-point Energy Correction = 0.24279 Hartree

Thermal Correction to Energy = 0.258016 Hartree

Thermal Correction to Enthalpy = 0.25896 Hartree

Thermal Correction to Free Energy = 0.199081 Hartree

EE + Zero-point Energy = -725.56151 Hartree

EE + Thermal Energy Correction = -725.54629 Hartree

EE + Thermal Enthalpy Correction = -725.54534 Hartree

EE + Thermal Free Energy Correction = -725.60522 Hartree

E (Thermal) = 161.907 kcal/mol

Heat Capacity (Cv) = 57.56 cal/mol-kelvin

Entropy (S) = 126.026 cal/mol-kelvin

TS1-en

Atom	Coordinates (Angstroms)		
	X	Y	Z
C	4.03381000	-0.36478400	-0.13643200
C	3.07904600	-0.43034100	-1.15746900
C	1.73366400	-0.18097900	-0.90637700
C	1.29430500	0.15353500	0.38190400
C	2.24579600	0.19149200	1.41108800
C	3.58985100	-0.05605500	1.15402700
H	3.39032300	-0.69544400	-2.16272 000
H	1.01730100	-0.27629900	-1.71347600
H	1.92761800	0.42180800	2.42235700
H	4.30382400	-0.01469800	1.97025300
C	-0.12746100	0.38606800	0.72260500
C	-1.37887900	-1.23434500	0.13135200
C	-2.52677000	-0.76648600	0.08268500
N	-0.89826100	1.34347000	0.17194500
O	-2.14540500	1.30460100	0.53869000
C	-0.65999900	1.92547900	-1.15041200
H	-1.04044700	1.25117800	-1.92458800
H	-1.21206600	2.86270300	-1.18903300
H	0.40204700	2.11258300	-1.29780400
H	-0.40276900	0.19544900	1.75246600
H	-0.72640400	-2.07950400	0.05469400
C	-3.99059600	-0.81641300	-0.07063100
N	-4.62268600	0.38403400	-0.10043700

The geometry of the transition state **TS1-en**

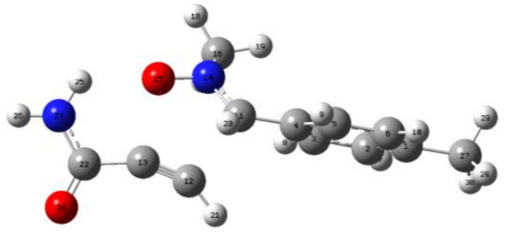

Electronic Energy (EE) = -725.7165 Hartree

Zero-point Energy Correction = 0.238377 Hartree

Thermal Correction to Energy = 0.254514 Hartree

Thermal Correction to Enthalpy = 0.255458 Hartree

Thermal Correction to Free Energy = 0.192273 Hartree

EE + Zero-point Energy = -725.47812 Hartree

EE + Thermal Energy Correction = -725.46199 Hartree

EE + Thermal Enthalpy Correction = -725.46104 Hartree

EE + Thermal Free Energy Correction = -725.52423 Hartree

E (Thermal) = 159.71 kcal/mol

Heat Capacity (Cv) = 59.852 cal/mol-kelvin

Entropy (S) = 132.983 cal/mol-kelvin

O	-4.56123600	-1.89186000	-0.17603000
H	-4.09972100	1.23199500	0.06206200
H	-5.62878800	0.38952300	-0.15024900
C	5.49537900	-0.60748400	-0.41942200
H	5.99782200	-1.06117000	0.43787000
H	6.01079700	0.33379300	-0.63997400
H	5.63133600	-1.26449300	-1.28119100

TS1-ex

Atom	Coordinates (Angstroms)		
	X	Y	Z
C	2.89907278	-0.18706532	-0.12146387
C	2.17468278	-1.09403332	0.66837713
C	0.81574278	-1.27579332	0.46371413
C	0.14875578	-0.56743232	-0.55110787
C	0.88510778	0.32923968	-1.34428987
C	2.24109778	0.52657468	-1.13278987
H	2.68463378	-1.64935532	1.44605813

The geometry of the transition state **TS1-ex**

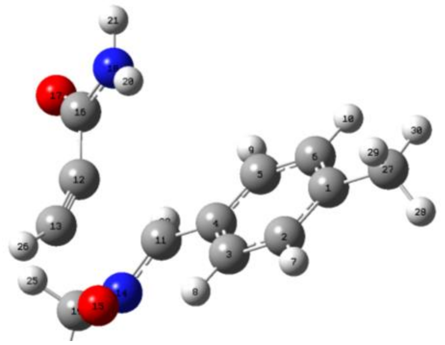

H	0.25911678	-1.96078332	1.08716513
H	0.38625778	0.87881568	-2.13490887
H	2.79585778	1.22361768	-1.74842487
C	-1.27375622	-0.73409232	-0.87526087
C	-1.95835649	0.71978987	0.73588029
C	-2.26176649	-0.10968213	1.61277229
N	-2.06871322	-1.68903732	-0.37913387
O	-2.02527522	-1.88330032	0.91580413
C	-1.91311449	2.06284887	0.17449329
O	-2.62221649	2.41056787	-0.75960171
N	-0.98091449	2.89021887	0.74028229
C	-3.38248422	-1.93290332	-0.98621487
H	-0.50088349	2.63260187	1.58671929
H	-0.95335349	3.84988187	0.43101229
H	-1.57526322	-0.36982932	-1.85134187
H	-3.69285222	-2.93987432	-0.71358387
H	-3.31124422	-1.84770732	-2.06984587
H	-4.09822722	-1.20530732	-0.59322087
H	-2.63788349	-0.42081513	2.56388529
C	4.32957307	-0.09364775	0.27402126
H	4.68622749	-1.10245776	0.27402126
H	4.68624591	0.41075044	1.14767277
H	4.68624591	0.41075044	-0.59963024

Electronic Energy (EE) = -725.71739 Hartree

Zero-point Energy Correction = 0.238299 Hartree

Thermal Correction to Energy = 0.254368 Hartree

Thermal Correction to Enthalpy = 0.255312 Hartree

Thermal Correction to Free Energy = 0.193518 Hartree

EE + Zero-point Energy = -725.47909 Hartree

EE + Thermal Energy Correction = -725.46302 Hartree

EE + Thermal Enthalpy Correction = -725.46208 Hartree

EE + Thermal Free Energy Correction = -725.52387 Hartree

E (Thermal) = 159.619 kcal/mol

Heat Capacity (Cv) = 59.925 cal/mol-kelvin

Entropy (S) = 130.058 cal/mol-kelvin

TS2-en

Atom	Coordinates (Angstroms)		
	X	Y	Z
C	-3.59426495	-0.58630386	-0.16283626
C	-3.43744595	0.42363114	0.78896074
C	-2.22554695	1.09418514	0.92794874
C	-1.12277295	0.76984214	0.12624174
C	-1.27250095	-0.25192786	-0.82601526
C	-2.49040095	-0.90256286	-0.96725626
H	-4.27300095	0.69623314	1.42528874
H	-2.13916495	1.88570914	1.66624874
H	-0.42857395	-0.52583986	-1.44268726
H	-2.58422595	-1.68481686	-1.71425426
C	0.11621005	1.54468514	0.33241474
C	1.21328405	0.14003314	1.50455274
C	1.94960605	-0.46394086	0.70790274
N	1.09598305	1.68095814	-0.58480026
O	1.54587505	0.58338914	-1.12139926
C	2.10426705	2.73015414	-0.40454026
H	2.55877905	2.92779314	-1.37401226
H	1.63673405	3.63971414	-0.02784026
H	2.87084705	2.37619714	0.29266674
H	0.82902105	0.23705114	2.49864474
C	2.90463205	-1.50654186	0.29783874
N	3.45950905	-1.34725386	-0.93149926
O	3.17056805	-2.43006386	1.05194174

The geometry of the transition state **TS2-en**

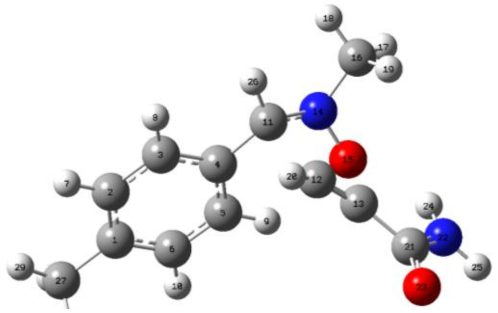

Electronic Energy (EE) = -725.7181 Hartree

Zero-point Energy Correction = 0.237742 Hartree

Thermal Correction to Energy = 0.25438 Hartree

Thermal Correction to Enthalpy = 0.255325 Hartree

Thermal Correction to Free Energy = 0.191479 Hartree

EE + Zero-point Energy = -725.48036 Hartree

EE + Thermal Energy Correction = -725.46372 Hartree

EE + Thermal Enthalpy Correction = -725.46277 Hartree

EE + Thermal Free Energy Correction = -725.52662 Hartree

E (Thermal) = 159.626 kcal/mol

Heat Capacity (Cv) = 60.365 cal/mol-kelvin

Entropy (S) = 134.374 cal/mol-kelvin

H	3.13444005	-0.60953086	-1.53869026
H	4.08332805	-2.06378086	-1.26668726
H	-0.01843795	2.43332514	0.94246174
C	-4.90251195	-1.31949986	-0.32458526
H	-4.77881295	-2.39169186	-0.14310626
H	-5.65707995	-0.94696186	0.37094574
H	-5.29576995	-1.20722086	-1.33976226

TS2-ex

Atom	Coordinates (Angstroms)		
	X	Y	Z
C	3.35130200	0.07823600	-0.05469600
C	2.90999800	-0.29446500	1.22011400
C	1.57516000	-0.60269700	1.45341200
C	0.63602700	-0.57032700	0.41092800
C	1.06864600	-0.16716100	-0.86061900
C	2.40454800	0.14671500	-1.08295300
H	3.61781000	-0.33541600	2.04166200
H	1.25541900	-0.88092300	2.45216400

The geometry of the transition state **TS2-ex**

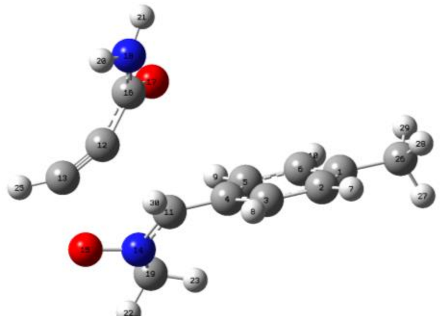

H	0.35062800	-0.03763900	-1.65983900
H	2.71147300	0.47494500	-2.07053400
C	-0.76379600	-0.89151600	0.72550400
C	-2.37833600	0.72674600	0.04828300
C	-3.33805400	-0.06806100	0.04941000
N	-1.50946000	-1.80623400	0.11206800
O	-2.78665200	-1.80307200	0.43426500
C	-1.74440400	1.99665500	-0.21265500
O	-1.18855200	2.25512100	-1.27449400
N	-1.74229600	2.88286300	0.84331700
C	-1.21531000	-2.38184900	-1.20152200
H	-2.40042200	2.76295300	1.59682400
H	-1.42217300	3.81827600	0.64106000
H	-1.74561400	-3.33058900	-1.25884000
H	-0.14397800	-2.54086100	-1.30790300
H	-1.57986100	-1.71267200	-1.98562500
H	-4.37505800	-0.30366500	-0.07308300
C	4.80341200	0.39311100	-0.31389600
H	5.34372100	-0.50210200	-0.64142800
H	5.29882200	0.76288200	0.58659200
H	4.91376700	1.14567800	-1.09790000
H	-1.11577400	-0.66598300	1.72312500

Electronic Energy (EE) = -725.71718 Hartree

Zero-point Energy Correction = 0.238416 Hartree

Thermal Correction to Energy = 0.25463 Hartree

Thermal Correction to Enthalpy = 0.255574 Hartree

Thermal Correction to Free Energy = 0.193433 Hartree

EE + Zero-point Energy = -725.47877 Hartree

EE + Thermal Energy Correction = -725.46255 Hartree

EE + Thermal Enthalpy Correction = -725.46161 Hartree

EE + Thermal Free Energy Correction = -725.52375 Hartree

E (Thermal) = 159.783 kcal/mol

Heat Capacity (Cv) = 59.966 cal/mol-kelvin

Entropy (S) = 130.786 cal/mol-kelvin

Wiberg bond index matrix in the NAO basis for TSs:

TS1-en

Atom		1	2	3	4	5	6	7	8	9
----		------	------	------	------	------	------	------	------	------
1.	C	0.0000	1.3811	0.0133	0.0989	0.0135	1.4075	0.0036	0.0085	0.0082
2.	C	1.3811	0.0000	1.4663	0.0129	0.1071	0.0122	0.9249	0.0039	0.0006
3.	C	0.0133	1.4663	0.0000	1.3589	0.0134	0.1030	0.0035	0.9234	0.0103
4.	C	0.0989	0.0129	1.3589	0.0000	1.3852	0.0128	0.0089	0.0032	0.0033
5.	C	0.0135	0.1071	0.0134	1.3852	0.0000	1.4375	0.0005	0.0100	0.9248
6.	C	1.4075	0.0122	0.1030	0.0128	1.4375	0.0000	0.0103	0.0006	0.0036
7.	H	0.0036	0.9249	0.0035	0.0089	0.0005	0.0103	0.0000	0.0034	0.0006
8.	H	0.0085	0.0039	0.9234	0.0032	0.0100	0.0006	0.0034	0.0000	0.0005
9.	H	0.0082	0.0006	0.0103	0.0033	0.9248	0.0036	0.0006	0.0005	0.0000
10.	H	0.0037	0.0105	0.0005	0.0085	0.0034	0.9249	0.0005	0.0006	0.0033
11.	C	0.0020	0.0093	0.0120	1.0578	0.0144	0.0089	0.0003	0.0017	0.0019
12.	C	0.0041	0.0004	0.0056	0.0034	0.0117	0.0005	0.0000	0.0006	0.0001
13.	C	0.0002	0.0002	0.0008	0.0032	0.0006	0.0007	0.0000	0.0002	0.0000
14.	N	0.0083	0.0010	0.0149	0.0101	0.0168	0.0006	0.0001	0.0003	0.0003
15.	O	0.0065	0.0005	0.0095	0.0148	0.0106	0.0008	0.0001	0.0004	0.0004
16.	C	0.0005	0.0011	0.0019	0.0012	0.0010	0.0005	0.0000	0.0005	0.0000
17.	H	0.0003	0.0001	0.0007	0.0002	0.0004	0.0000	0.0000	0.0002	0.0000
18.	H	0.0002	0.0001	0.0005	0.0003	0.0003	0.0002	0.0000	0.0001	0.0000
19.	H	0.0001	0.0002	0.0016	0.0006	0.0002	0.0001	0.0000	0.0000	0.0000
20.	H	0.0009	0.0002	0.0085	0.0024	0.0021	0.0004	0.0003	0.0002	0.0003
21.	H	0.0000	0.0000	0.0005	0.0003	0.0001	0.0000	0.0000	0.0000	0.0000
22.	C	0.0002	0.0000	0.0003	0.0001	0.0004	0.0000	0.0000	0.0000	0.0000
23.	N	0.0001	0.0000	0.0001	0.0001	0.0001	0.0000	0.0000	0.0000	0.0000
24.	O	0.0000	0.0000	0.0001	0.0003	0.0000	0.0001	0.0000	0.0000	0.0000

25.	H	0.0000	0.0000	0.0000	0.0001	0.0000	0.0000	0.0000	0.0000	0.0000
26.	H	0.0000	0.0000	0.0000	0.0000	0.0000	0.0000	0.0000	0.0000	0.0000
27.	C	1.0352	0.0110	0.0097	0.0015	0.0088	0.0109	0.0027	0.0004	0.0004
28.	H	0.0020	0.0074	0.0003	0.0001	0.0005	0.0019	0.0004	0.0003	0.0000
29.	H	0.0025	0.0058	0.0002	0.0038	0.0002	0.0124	0.0001	0.0000	0.0001
30.	H	0.0025	0.0067	0.0002	0.0044	0.0002	0.0126	0.0001	0.0000	0.0001

Atom		10	11	12	13	14	15	16	17	18
1.	C	0.0037	0.0020	0.0041	0.0002	0.0083	0.0065	0.0005	0.0003	0.0002
2.	C	0.0105	0.0093	0.0004	0.0002	0.0010	0.0005	0.0011	0.0001	0.0001
3.	C	0.0005	0.0120	0.0056	0.0008	0.0149	0.0095	0.0019	0.0007	0.0005
4.	C	0.0085	1.0578	0.0034	0.0032	0.0101	0.0148	0.0012	0.0002	0.0003
5.	C	0.0034	0.0144	0.0117	0.0006	0.0168	0.0106	0.0010	0.0004	0.0003
6.	C	0.9249	0.0089	0.0005	0.0007	0.0006	0.0008	0.0005	0.0000	0.0002
7.	H	0.0005	0.0003	0.0000	0.0000	0.0001	0.0001	0.0000	0.0000	0.0000
8.	H	0.0006	0.0017	0.0006	0.0002	0.0003	0.0004	0.0005	0.0002	0.0001
9.	H	0.0033	0.0019	0.0001	0.0000	0.0003	0.0004	0.0000	0.0000	0.0000
10.	H	0.0000	0.0002	0.0001	0.0000	0.0002	0.0002	0.0000	0.0000	0.0000
11.	C	0.0002	0.0000	0.3863	0.0115	1.2939	0.1842	0.0169	0.0103	0.0082
12.	C	0.0001	0.3863	0.0000	2.4346	0.0345	0.0294	0.0012	0.0028	0.0005
13.	C	0.0000	0.0115	2.4346	0.0000	0.1063	0.2960	0.0013	0.0077	0.0017
14.	N	0.0002	1.2939	0.0345	0.1063	0.0000	1.1957	0.9385	0.0057	0.0029
15.	O	0.0002	0.1842	0.0294	0.2960	1.1957	0.0000	0.0469	0.0027	0.0026
16.	C	0.0000	0.0169	0.0012	0.0013	0.9385	0.0469	0.0000	0.9295	0.9364
17.	H	0.0000	0.0103	0.0028	0.0077	0.0057	0.0027	0.9295	0.0000	0.0006
18.	H	0.0000	0.0082	0.0005	0.0017	0.0029	0.0026	0.9364	0.0006	0.0000
19.	H	0.0000	0.0008	0.0001	0.0005	0.0026	0.0127	0.9378	0.0004	0.0007

20.	H	0.0000	0.9034	0.0018	0.0086	0.0031	0.0037	0.0128	0.0001	0.0002
21.	H	0.0000	0.0009	0.9023	0.0129	0.0059	0.0201	0.0001	0.0004	0.0001
22.	C	0.0000	0.0085	0.0229	1.0105	0.0017	0.0071	0.0002	0.0001	0.0000
23.	N	0.0000	0.0009	0.0328	0.0126	0.0029	0.0075	0.0004	0.0001	0.0001
24.	O	0.0000	0.0007	0.0616	0.0810	0.0064	0.0162	0.0003	0.0005	0.0002
25.	H	0.0000	0.0003	0.0009	0.0005	0.0004	0.0118	0.0001	0.0000	0.0000
26.	H	0.0000	0.0001	0.0009	0.0133	0.0004	0.0014	0.0000	0.0000	0.0000
27.	C	0.0026	0.0006	0.0000	0.0000	0.0002	0.0001	0.0000	0.0000	0.0000
28.	H	0.0004	0.0000	0.0000	0.0000	0.0000	0.0000	0.0000	0.0000	0.0000
29.	H	0.0002	0.0001	0.0002	0.0000	0.0004	0.0004	0.0000	0.0000	0.0000
30.	H	0.0001	0.0001	0.0002	0.0000	0.0004	0.0003	0.0000	0.0000	0.0000

Atom		19	20	21	22	23	24	25	26	27
----		------	------	------	------	------	------	------	------	------
1.	C	0.0001	0.0009	0.0000	0.0002	0.0001	0.0000	0.0000	0.0000	1.0352
2.	C	0.0002	0.0002	0.0000	0.0000	0.0000	0.0000	0.0000	0.0000	0.0110
3.	C	0.0016	0.0085	0.0005	0.0003	0.0001	0.0001	0.0000	0.0000	0.0097
4.	C	0.0006	0.0024	0.0003	0.0001	0.0001	0.0003	0.0001	0.0000	0.0015
5.	C	0.0002	0.0021	0.0001	0.0004	0.0001	0.0000	0.0000	0.0000	0.0088
6.	C	0.0001	0.0004	0.0000	0.0000	0.0000	0.0001	0.0000	0.0000	0.0109
7.	H	0.0000	0.0003	0.0000	0.0000	0.0000	0.0000	0.0000	0.0000	0.0027
8.	H	0.0000	0.0002	0.0000	0.0000	0.0000	0.0000	0.0000	0.0000	0.0004
9.	H	0.0000	0.0003	0.0000	0.0000	0.0000	0.0000	0.0000	0.0000	0.0004
10.	H	0.0000	0.0000	0.0000	0.0000	0.0000	0.0000	0.0000	0.0000	0.0026
11.	C	0.0008	0.9034	0.0009	0.0085	0.0009	0.0007	0.0003	0.0001	0.0006
12.	C	0.0001	0.0018	0.9023	0.0229	0.0328	0.0616	0.0009	0.0009	0.0000
13.	C	0.0005	0.0086	0.0129	1.0105	0.0126	0.0810	0.0005	0.0133	0.0000
14.	N	0.0026	0.0031	0.0059	0.0017	0.0029	0.0064	0.0004	0.0004	0.0002

15.	O	0.0127	0.0037	0.0201	0.0071	0.0075	0.0162	0.0118	0.0014	0.0001
16.	C	0.9378	0.0128	0.0001	0.0002	0.0004	0.0003	0.0001	0.0000	0.0000
17.	H	0.0004	0.0001	0.0004	0.0001	0.0001	0.0005	0.0000	0.0000	0.0000
18.	H	0.0007	0.0002	0.0001	0.0000	0.0001	0.0002	0.0000	0.0000	0.0000
19.	H	0.0000	0.0003	0.0000	0.0000	0.0000	0.0001	0.0000	0.0000	0.0000
20.	H	0.0003	0.0000	0.0003	0.0003	0.0002	0.0008	0.0000	0.0000	0.0000
21.	H	0.0000	0.0003	0.0000	0.0018	0.0001	0.0012	0.0000	0.0001	0.0000
22.	C	0.0000	0.0003	0.0018	0.0000	1.2231	1.6386	0.0033	0.0020	0.0000
23.	N	0.0000	0.0002	0.0001	1.2231	0.0000	0.1932	0.7958	0.8273	0.0000
24.	O	0.0001	0.0008	0.0012	1.6386	0.1932	0.0000	0.0119	0.0033	0.0000
25.	H	0.0000	0.0000	0.0000	0.0033	0.7958	0.0119	0.0000	0.0003	0.0000
26.	H	0.0000	0.0000	0.0001	0.0020	0.8273	0.0033	0.0003	0.0000	0.0000
27.	C	0.0000	0.0000	0.0000	0.0000	0.0000	0.0000	0.0000	0.0000	0.0000
28.	H	0.0000	0.0000	0.0000	0.0000	0.0000	0.0000	0.0000	0.0000	0.9441
29.	H	0.0000	0.0000	0.0000	0.0000	0.0000	0.0000	0.0000	0.0000	0.9285
30.	H	0.0000	0.0001	0.0000	0.0000	0.0000	0.0000	0.0000	0.0000	0.9266

Atom		28	29	30
1.	C	0.0020	0.0025	0.0025
2.	C	0.0074	0.0058	0.0067
3.	C	0.0003	0.0002	0.0002
4.	C	0.0001	0.0038	0.0044
5.	C	0.0005	0.0002	0.0002
6.	C	0.0019	0.0124	0.0126
7.	H	0.0004	0.0001	0.0001
8.	H	0.0003	0.0000	0.0000
9.	H	0.0000	0.0001	0.0001

10.	H	0.0004	0.0002	0.0001
11.	C	0.0000	0.0001	0.0001
12.	C	0.0000	0.0002	0.0002
13.	C	0.0000	0.0000	0.0000
14.	N	0.0000	0.0004	0.0004
15.	O	0.0000	0.0004	0.0003
16.	C	0.0000	0.0000	0.0000
17.	H	0.0000	0.0000	0.0000
18.	H	0.0000	0.0000	0.0000
19.	H	0.0000	0.0000	0.0000
20.	H	0.0000	0.0000	0.0001
21.	H	0.0000	0.0000	0.0000
22.	C	0.0000	0.0000	0.0000
23.	N	0.0000	0.0000	0.0000
24.	O	0.0000	0.0000	0.0000
25.	H	0.0000	0.0000	0.0000
26.	H	0.0000	0.0000	0.0000
27.	C	0.9441	0.9285	0.9266
28.	H	0.0000	0.0006	0.0006
29.	H	0.0006	0.0000	0.0007
30.	H	0.0006	0.0007	0.0000

Wiberg bond index matrix in the NAO basis:

TS1-ex

Atom		1	2	3	4	5	6	7	8	9
1.	C	0.0000	1.3744	0.0136	0.0920	0.0133	1.4045	0.0035	0.0081	0.0084
2.	C	1.3744	0.0000	1.4768	0.0121	0.1020	0.0120	0.9258	0.0039	0.0006

3.	C	0.0136	1.4768	0.0000	1.3341	0.0130	0.1015	0.0037	0.9106	0.0104
4.	C	0.0920	0.0121	1.3341	0.0000	1.3670	0.0130	0.0089	0.0032	0.0033
5.	C	0.0133	0.1020	0.0130	1.3670	0.0000	1.4415	0.0006	0.0095	0.9264
6.	C	1.4045	0.0120	0.1015	0.0130	1.4415	0.0000	0.0104	0.0005	0.0039
7.	H	0.0035	0.9258	0.0037	0.0089	0.0006	0.0104	0.0000	0.0034	0.0006
8.	H	0.0081	0.0039	0.9106	0.0032	0.0095	0.0005	0.0034	0.0000	0.0005
9.	H	0.0084	0.0006	0.0104	0.0033	0.9264	0.0039	0.0006	0.0005	0.0000
10.	H	0.0038	0.0106	0.0006	0.0088	0.0035	0.9257	0.0005	0.0005	0.0034
11.	C	0.0022	0.0112	0.0114	1.1069	0.0130	0.0096	0.0002	0.0015	0.0019
12.	C	0.0106	0.0008	0.0153	0.0053	0.0157	0.0002	0.0000	0.0002	0.0001
13.	C	0.0012	0.0003	0.0009	0.0027	0.0028	0.0002	0.0000	0.0002	0.0000
14.	N	0.0115	0.0007	0.0141	0.0141	0.0237	0.0007	0.0000	0.0002	0.0004
15.	O	0.0097	0.0016	0.0166	0.0036	0.0124	0.0004	0.0002	0.0043	0.0000
16.	C	0.0005	0.0001	0.0011	0.0013	0.0008	0.0001	0.0000	0.0000	0.0001
17.	O	0.0012	0.0001	0.0017	0.0008	0.0016	0.0001	0.0000	0.0000	0.0001
18.	N	0.0008	0.0002	0.0007	0.0009	0.0031	0.0008	0.0000	0.0000	0.0002
19.	C	0.0003	0.0003	0.0008	0.0113	0.0008	0.0003	0.0000	0.0001	0.0001
20.	H	0.0000	0.0001	0.0000	0.0001	0.0000	0.0000	0.0000	0.0000	0.0000
21.	H	0.0000	0.0000	0.0000	0.0000	0.0000	0.0000	0.0000	0.0000	0.0000
22.	H	0.0005	0.0001	0.0086	0.0026	0.0019	0.0004	0.0004	0.0002	0.0004
23.	H	0.0002	0.0000	0.0002	0.0004	0.0004	0.0000	0.0000	0.0000	0.0000
24.	H	0.0000	0.0000	0.0001	0.0004	0.0001	0.0000	0.0000	0.0000	0.0000
25.	H	0.0007	0.0000	0.0008	0.0002	0.0010	0.0000	0.0000	0.0000	0.0000
26.	H	0.0009	0.0000	0.0011	0.0001	0.0012	0.0000	0.0000	0.0000	0.0000
27.	C	1.0355	0.0109	0.0098	0.0013	0.0088	0.0106	0.0026	0.0004	0.0004
28.	H	0.0025	0.0061	0.0002	0.0039	0.0002	0.0126	0.0001	0.0000	0.0001
29.	H	0.0025	0.0060	0.0002	0.0038	0.0002	0.0124	0.0001	0.0000	0.0001
30.	H	0.0020	0.0075	0.0003	0.0000	0.0005	0.0019	0.0004	0.0003	0.0001

Atom		10	11	12	13	14	15	16	17	18
----		------	------	------	------	------	------	------	------	------
1.	C	0.0038	0.0022	0.0106	0.0012	0.0115	0.0097	0.0005	0.0012	0.0008
2.	C	0.0106	0.0112	0.0008	0.0003	0.0007	0.0016	0.0001	0.0001	0.0002
3.	C	0.0006	0.0114	0.0153	0.0009	0.0141	0.0166	0.0011	0.0017	0.0007
4.	C	0.0088	1.1069	0.0053	0.0027	0.0141	0.0036	0.0013	0.0008	0.0009
5.	C	0.0035	0.0130	0.0157	0.0028	0.0237	0.0124	0.0008	0.0016	0.0031
6.	C	0.9257	0.0096	0.0002	0.0002	0.0007	0.0004	0.0001	0.0001	0.0008
7.	H	0.0005	0.0002	0.0000	0.0000	0.0000	0.0002	0.0000	0.0000	0.0000
8.	H	0.0005	0.0015	0.0002	0.0002	0.0002	0.0043	0.0000	0.0000	0.0000
9.	H	0.0034	0.0019	0.0001	0.0000	0.0004	0.0000	0.0001	0.0001	0.0002
10.	H	0.0000	0.0002	0.0000	0.0000	0.0002	0.0000	0.0000	0.0000	0.0001
11.	C	0.0002	0.0000	0.2698	0.0039	1.3331	0.1644	0.0064	0.0114	0.0110
12.	C	0.0000	0.2698	0.0000	2.3877	0.0462	0.0457	1.0588	0.0829	0.0172
13.	C	0.0000	0.0039	2.3877	0.0000	0.0618	0.4287	0.0283	0.0560	0.0230
14.	N	0.0002	1.3331	0.0462	0.0618	0.0000	1.1485	0.0056	0.0058	0.0043
15.	O	0.0000	0.1644	0.0457	0.4287	1.1485	0.0000	0.0153	0.0075	0.0032
16.	C	0.0000	0.0064	1.0588	0.0283	0.0056	0.0153	0.0000	1.6225	1.1713
17.	O	0.0000	0.0114	0.0829	0.0560	0.0058	0.0075	1.6225	0.0000	0.1697
18.	N	0.0001	0.0110	0.0172	0.0230	0.0043	0.0032	1.1713	0.1697	0.0000
19.	C	0.0001	0.0143	0.0022	0.0014	0.9377	0.0428	0.0003	0.0005	0.0001
20.	H	0.0000	0.0001	0.0008	0.0007	0.0000	0.0001	0.0030	0.0143	0.8285
21.	H	0.0000	0.0001	0.0119	0.0026	0.0001	0.0007	0.0021	0.0036	0.8260
22.	H	0.0000	0.9199	0.0007	0.0005	0.0037	0.0140	0.0001	0.0003	0.0001
23.	H	0.0000	0.0088	0.0007	0.0010	0.0027	0.0019	0.0001	0.0002	0.0001
24.	H	0.0000	0.0004	0.0002	0.0003	0.0029	0.0127	0.0000	0.0001	0.0000
25.	H	0.0000	0.0102	0.0031	0.0044	0.0054	0.0024	0.0003	0.0003	0.0002

26.	H	0.0000	0.0151	0.0115	0.9108	0.0020	0.0089	0.0027	0.0010	0.0003
27.	C	0.0025	0.0007	0.0001	0.0000	0.0002	0.0002	0.0000	0.0000	0.0000
28.	H	0.0001	0.0001	0.0005	0.0001	0.0006	0.0005	0.0000	0.0001	0.0000
29.	H	0.0002	0.0001	0.0005	0.0001	0.0006	0.0005	0.0000	0.0001	0.0001
30.	H	0.0005	0.0000	0.0000	0.0000	0.0000	0.0000	0.0000	0.0000	0.0000

Atom		19	20	21	22	23	24	25	26	27
----		------	------	------	------	------	------	------	------	------
1.	C	0.0003	0.0000	0.0000	0.0005	0.0002	0.0000	0.0007	0.0009	1.0355
2.	C	0.0003	0.0001	0.0000	0.0001	0.0000	0.0000	0.0000	0.0000	0.0109
3.	C	0.0008	0.0000	0.0000	0.0086	0.0002	0.0001	0.0008	0.0011	0.0098
4.	C	0.0113	0.0001	0.0000	0.0026	0.0004	0.0004	0.0002	0.0001	0.0013
5.	C	0.0008	0.0000	0.0000	0.0019	0.0004	0.0001	0.0010	0.0012	0.0088
6.	C	0.0003	0.0000	0.0000	0.0004	0.0000	0.0000	0.0000	0.0000	0.0106
7.	H	0.0000	0.0000	0.0000	0.0004	0.0000	0.0000	0.0000	0.0000	0.0026
8.	H	0.0001	0.0000	0.0000	0.0002	0.0000	0.0000	0.0000	0.0000	0.0004
9.	H	0.0001	0.0000	0.0000	0.0004	0.0000	0.0000	0.0000	0.0000	0.0004
10.	H	0.0001	0.0000	0.0000	0.0000	0.0000	0.0000	0.0000	0.0000	0.0025
11.	C	0.0143	0.0001	0.0001	0.9199	0.0088	0.0004	0.0102	0.0151	0.0007
12.	C	0.0022	0.0008	0.0119	0.0007	0.0007	0.0002	0.0031	0.0115	0.0001
13.	C	0.0014	0.0007	0.0026	0.0005	0.0010	0.0003	0.0044	0.9108	0.0000
14.	N	0.9377	0.0000	0.0001	0.0037	0.0027	0.0029	0.0054	0.0020	0.0002
15.	O	0.0428	0.0001	0.0007	0.0140	0.0019	0.0127	0.0024	0.0089	0.0002
16.	C	0.0003	0.0030	0.0021	0.0001	0.0001	0.0000	0.0003	0.0027	0.0000
17.	O	0.0005	0.0143	0.0036	0.0003	0.0002	0.0001	0.0003	0.0010	0.0000
18.	N	0.0001	0.8285	0.8260	0.0001	0.0001	0.0000	0.0002	0.0003	0.0000
19.	C	0.0000	0.0000	0.0000	0.0012	0.9380	0.9443	0.9266	0.0001	0.0000
20.	H	0.0000	0.0000	0.0002	0.0000	0.0000	0.0000	0.0000	0.0000	0.0000

21. H	0.0000	0.0002	0.0000	0.0000	0.0000	0.0000	0.0000	0.0000	0.0000
22. H	0.0012	0.0000	0.0000	0.0000	0.0005	0.0001	0.0001	0.0000	0.0000
23. H	0.9380	0.0000	0.0000	0.0005	0.0000	0.0007	0.0005	0.0000	0.0000
24. H	0.9443	0.0000	0.0000	0.0001	0.0007	0.0000	0.0005	0.0000	0.0000
25. H	0.9266	0.0000	0.0000	0.0001	0.0005	0.0005	0.0000	0.0001	0.0000
26. H	0.0001	0.0000	0.0000	0.0000	0.0000	0.0000	0.0001	0.0000	0.0000
27. C	0.0000	0.0000	0.0000	0.0000	0.0000	0.0000	0.0000	0.0000	0.0000
28. H	0.0000	0.0000	0.0000	0.0000	0.0000	0.0000	0.0000	0.0000	0.9276
29. H	0.0000	0.0000	0.0000	0.0000	0.0000	0.0000	0.0000	0.0000	0.9286
30. H	0.0000	0.0000	0.0000	0.0000	0.0000	0.0000	0.0000	0.0000	0.9450

Atom	28	29	30
1. C	0.0025	0.0025	0.0020
2. C	0.0061	0.0060	0.0075
3. C	0.0002	0.0002	0.0003
4. C	0.0039	0.0038	0.0000
5. C	0.0002	0.0002	0.0005
6. C	0.0126	0.0124	0.0019
7. H	0.0001	0.0001	0.0004
8. H	0.0000	0.0000	0.0003
9. H	0.0001	0.0001	0.0001
10. H	0.0001	0.0002	0.0005
11. C	0.0001	0.0001	0.0000
12. C	0.0005	0.0005	0.0000
13. C	0.0001	0.0001	0.0000
14. N	0.0006	0.0006	0.0000
15. O	0.0005	0.0005	0.0000

16.	C	0.0000	0.0000	0.0000
17.	O	0.0001	0.0001	0.0000
18.	N	0.0000	0.0001	0.0000
19.	C	0.0000	0.0000	0.0000
20.	H	0.0000	0.0000	0.0000
21.	H	0.0000	0.0000	0.0000
22.	H	0.0000	0.0000	0.0000
23.	H	0.0000	0.0000	0.0000
24.	H	0.0000	0.0000	0.0000
25.	H	0.0000	0.0000	0.0000
26.	H	0.0000	0.0000	0.0000
27.	C	0.9276	0.9286	0.9450
28.	H	0.0000	0.0007	0.0006
29.	H	0.0007	0.0000	0.0006
30.	H	0.0006	0.0006	0.0000

Wiberg bond index matrix in the NAO basis:

TS2-en

Wiberg bond index matrix in the NAO basis:

Atom		1	2	3	4	5	6	7	8	9
----		------	------	------	------	------	------	------	------	------
1.	C	0.0000	1.4054	0.0131	0.0941	0.0138	1.3776	0.0038	0.0085	0.0080
2.	C	1.4054	0.0000	1.4389	0.0138	0.1026	0.0121	0.9261	0.0041	0.0005
3.	C	0.0131	1.4389	0.0000	1.3757	0.0132	0.1040	0.0034	0.9288	0.0094
4.	C	0.0941	0.0138	1.3757	0.0000	1.3423	0.0123	0.0086	0.0034	0.0033
5.	C	0.0138	0.1026	0.0132	1.3423	0.0000	1.4723	0.0006	0.0106	0.9094
6.	C	1.3776	0.0121	0.1040	0.0123	1.4723	0.0000	0.0106	0.0006	0.0039

7.	H	0.0038	0.9261	0.0034	0.0086	0.0006	0.0106	0.0000	0.0033	0.0005
8.	H	0.0085	0.0041	0.9288	0.0034	0.0106	0.0006	0.0033	0.0000	0.0005
9.	H	0.0080	0.0005	0.0094	0.0033	0.9094	0.0039	0.0005	0.0005	0.0000
10.	H	0.0034	0.0104	0.0006	0.0089	0.0037	0.9256	0.0005	0.0006	0.0034
11.	C	0.0019	0.0093	0.0126	1.0814	0.0114	0.0102	0.0003	0.0019	0.0015
12.	C	0.0073	0.0002	0.0129	0.0081	0.0117	0.0010	0.0001	0.0002	0.0001
13.	C	0.0024	0.0005	0.0048	0.0080	0.0021	0.0005	0.0001	0.0001	0.0003
14.	N	0.0104	0.0007	0.0215	0.0126	0.0129	0.0005	0.0002	0.0003	0.0002
15.	O	0.0098	0.0004	0.0122	0.0039	0.0177	0.0017	0.0000	0.0001	0.0039
16.	C	0.0004	0.0003	0.0008	0.0109	0.0009	0.0003	0.0001	0.0001	0.0001
17.	H	0.0001	0.0000	0.0003	0.0004	0.0002	0.0000	0.0000	0.0000	0.0000
18.	H	0.0000	0.0000	0.0001	0.0004	0.0001	0.0000	0.0000	0.0000	0.0000
19.	H	0.0007	0.0000	0.0009	0.0002	0.0008	0.0000	0.0000	0.0000	0.0000
20.	H	0.0000	0.0001	0.0002	0.0004	0.0001	0.0000	0.0000	0.0000	0.0000
21.	C	0.0004	0.0001	0.0005	0.0006	0.0006	0.0001	0.0000	0.0000	0.0000
22.	N	0.0002	0.0000	0.0003	0.0002	0.0003	0.0000	0.0000	0.0000	0.0001
23.	O	0.0003	0.0001	0.0004	0.0007	0.0003	0.0001	0.0000	0.0000	0.0000
24.	H	0.0000	0.0000	0.0000	0.0000	0.0000	0.0000	0.0000	0.0000	0.0000
25.	H	0.0000	0.0000	0.0000	0.0000	0.0000	0.0000	0.0000	0.0000	0.0000
26.	H	0.0004	0.0005	0.0019	0.0026	0.0084	0.0001	0.0001	0.0005	0.0002
27.	C	1.0352	0.0106	0.0088	0.0013	0.0098	0.0109	0.0025	0.0005	0.0004
28.	H	0.0025	0.0125	0.0002	0.0040	0.0002	0.0062	0.0002	0.0001	0.0000
29.	H	0.0020	0.0019	0.0005	0.0000	0.0003	0.0075	0.0005	0.0001	0.0003
30.	H	0.0025	0.0125	0.0002	0.0039	0.0002	0.0060	0.0001	0.0001	0.0000

Atom		10	11	12	13	14	15	16	17	18
1.	C	0.0034	0.0019	0.0073	0.0024	0.0104	0.0098	0.0004	0.0001	0.0000

2.	C	0.0104	0.0093	0.0002	0.0005	0.0007	0.0004	0.0003	0.0000	0.0000
3.	C	0.0006	0.0126	0.0129	0.0048	0.0215	0.0122	0.0008	0.0003	0.0001
4.	C	0.0089	1.0814	0.0081	0.0080	0.0126	0.0039	0.0109	0.0004	0.0004
5.	C	0.0037	0.0114	0.0117	0.0021	0.0129	0.0177	0.0009	0.0002	0.0001
6.	C	0.9256	0.0102	0.0010	0.0005	0.0005	0.0017	0.0003	0.0000	0.0000
7.	H	0.0005	0.0003	0.0001	0.0001	0.0002	0.0000	0.0001	0.0000	0.0000
8.	H	0.0006	0.0019	0.0002	0.0001	0.0003	0.0001	0.0001	0.0000	0.0000
9.	H	0.0034	0.0015	0.0001	0.0003	0.0002	0.0039	0.0001	0.0000	0.0000
10.	H	0.0000	0.0002	0.0000	0.0000	0.0001	0.0002	0.0000	0.0000	0.0000
11.	C	0.0002	0.0000	0.3786	0.0085	1.2746	0.1780	0.0127	0.0081	0.0004
12.	C	0.0000	0.3786	0.0000	2.4286	0.0344	0.0295	0.0014	0.0005	0.0001
13.	C	0.0000	0.0085	2.4286	0.0000	0.1060	0.3010	0.0012	0.0020	0.0002
14.	N	0.0001	1.2746	0.0344	0.1060	0.0000	1.1852	0.9395	0.0029	0.0028
15.	O	0.0002	0.1780	0.0295	0.3010	1.1852	0.0000	0.0488	0.0019	0.0132
16.	C	0.0000	0.0127	0.0014	0.0012	0.9395	0.0488	0.0000	0.9381	0.9451
17.	H	0.0000	0.0081	0.0005	0.0020	0.0029	0.0019	0.9381	0.0000	0.0007
18.	H	0.0000	0.0004	0.0001	0.0002	0.0028	0.0132	0.9451	0.0007	0.0000
19.	H	0.0000	0.0093	0.0024	0.0075	0.0061	0.0022	0.9280	0.0005	0.0004
20.	H	0.0000	0.0008	0.9033	0.0130	0.0054	0.0197	0.0001	0.0001	0.0000
21.	C	0.0000	0.0085	0.0228	1.0120	0.0017	0.0072	0.0003	0.0000	0.0000
22.	N	0.0000	0.0007	0.0326	0.0129	0.0023	0.0089	0.0003	0.0001	0.0000
23.	O	0.0000	0.0005	0.0616	0.0819	0.0061	0.0171	0.0002	0.0002	0.0000
24.	H	0.0000	0.0003	0.0009	0.0005	0.0004	0.0109	0.0001	0.0000	0.0000
25.	H	0.0000	0.0001	0.0009	0.0132	0.0004	0.0013	0.0000	0.0000	0.0000
26.	H	0.0004	0.9224	0.0005	0.0009	0.0035	0.0148	0.0011	0.0005	0.0001
27.	C	0.0026	0.0006	0.0001	0.0001	0.0002	0.0002	0.0000	0.0000	0.0000
28.	H	0.0001	0.0001	0.0004	0.0001	0.0005	0.0005	0.0000	0.0000	0.0000
29.	H	0.0004	0.0000	0.0000	0.0000	0.0000	0.0000	0.0000	0.0000	0.0000

30.	H	0.0001	0.0001	0.0003	0.0001	0.0005	0.0005	0.0000	0.0000	0.0000

Atom		19	20	21	22	23	24	25	26	27
----		------	------	------	------	------	------	------	------	------
1.	C	0.0007	0.0000	0.0004	0.0002	0.0003	0.0000	0.0000	0.0004	1.0352
2.	C	0.0000	0.0001	0.0001	0.0000	0.0001	0.0000	0.0000	0.0005	0.0106
3.	C	0.0009	0.0002	0.0005	0.0003	0.0004	0.0000	0.0000	0.0019	0.0088
4.	C	0.0002	0.0004	0.0006	0.0002	0.0007	0.0000	0.0000	0.0026	0.0013
5.	C	0.0008	0.0001	0.0006	0.0003	0.0003	0.0000	0.0000	0.0084	0.0098
6.	C	0.0000	0.0000	0.0001	0.0000	0.0001	0.0000	0.0000	0.0001	0.0109
7.	H	0.0000	0.0000	0.0000	0.0000	0.0000	0.0000	0.0000	0.0001	0.0025
8.	H	0.0000	0.0000	0.0000	0.0000	0.0000	0.0000	0.0000	0.0005	0.0005
9.	H	0.0000	0.0000	0.0000	0.0001	0.0000	0.0000	0.0000	0.0002	0.0004
10.	H	0.0000	0.0000	0.0000	0.0000	0.0000	0.0000	0.0000	0.0004	0.0026
11.	C	0.0093	0.0008	0.0085	0.0007	0.0005	0.0003	0.0001	0.9224	0.0006
12.	C	0.0024	0.9033	0.0228	0.0326	0.0616	0.0009	0.0009	0.0005	0.0001
13.	C	0.0075	0.0130	1.0120	0.0129	0.0819	0.0005	0.0132	0.0009	0.0001
14.	N	0.0061	0.0054	0.0017	0.0023	0.0061	0.0004	0.0004	0.0035	0.0002
15.	O	0.0022	0.0197	0.0072	0.0089	0.0171	0.0109	0.0013	0.0148	0.0002
16.	C	0.9280	0.0001	0.0003	0.0003	0.0002	0.0001	0.0000	0.0011	0.0000
17.	H	0.0005	0.0001	0.0000	0.0001	0.0002	0.0000	0.0000	0.0005	0.0000
18.	H	0.0004	0.0000	0.0000	0.0000	0.0000	0.0000	0.0000	0.0001	0.0000
19.	H	0.0000	0.0004	0.0001	0.0001	0.0004	0.0000	0.0000	0.0001	0.0000
20.	H	0.0004	0.0000	0.0018	0.0001	0.0013	0.0000	0.0001	0.0000	0.0000
21.	C	0.0001	0.0018	0.0000	1.2195	1.6406	0.0032	0.0019	0.0000	0.0000
22.	N	0.0001	0.0001	1.2195	0.0000	0.1917	0.7972	0.8270	0.0001	0.0000
23.	O	0.0004	0.0013	1.6406	0.1917	0.0000	0.0119	0.0033	0.0001	0.0000
24.	H	0.0000	0.0000	0.0032	0.7972	0.0119	0.0000	0.0002	0.0000	0.0000

25. H	0.0000	0.0001	0.0019	0.8270	0.0033	0.0002	0.0000	0.0000	0.0000
26. H	0.0001	0.0000	0.0000	0.0001	0.0001	0.0000	0.0000	0.0000	0.0000
27. C	0.0000	0.0000	0.0000	0.0000	0.0000	0.0000	0.0000	0.0000	0.0000
28. H	0.0000	0.0000	0.0000	0.0000	0.0000	0.0000	0.0000	0.0000	0.9274
29. H	0.0000	0.0000	0.0000	0.0000	0.0000	0.0000	0.0000	0.0000	0.9453
30. H	0.0000	0.0000	0.0000	0.0000	0.0000	0.0000	0.0000	0.0000	0.9285

Atom	28	29	30
1. C	0.0025	0.0020	0.0025
2. C	0.0125	0.0019	0.0125
3. C	0.0002	0.0005	0.0002
4. C	0.0040	0.0000	0.0039
5. C	0.0002	0.0003	0.0002
6. C	0.0062	0.0075	0.0060
7. H	0.0002	0.0005	0.0001
8. H	0.0001	0.0001	0.0001
9. H	0.0000	0.0003	0.0000
10. H	0.0001	0.0004	0.0001
11. C	0.0001	0.0000	0.0001
12. C	0.0004	0.0000	0.0003
13. C	0.0001	0.0000	0.0001
14. N	0.0005	0.0000	0.0005
15. O	0.0005	0.0000	0.0005
16. C	0.0000	0.0000	0.0000
17. H	0.0000	0.0000	0.0000
18. H	0.0000	0.0000	0.0000
19. H	0.0000	0.0000	0.0000

20.	H	0.0000	0.0000	0.0000
21.	C	0.0000	0.0000	0.0000
22.	N	0.0000	0.0000	0.0000
23.	O	0.0000	0.0000	0.0000
24.	H	0.0000	0.0000	0.0000
25.	H	0.0000	0.0000	0.0000
26.	H	0.0000	0.0000	0.0000
27.	C	0.9274	0.9453	0.9285
28.	H	0.0000	0.0006	0.0007
29.	H	0.0006	0.0000	0.0006
30.	H	0.0007	0.0006	0.0000

Wiberg bond index matrix in the NAO basis:

TS2-ex

Atom		1	2	3	4	5	6	7	8	9
----		------	------	------	------	------	------	------	------	------
1.	C	0.0000	1.3917	0.0134	0.0964	0.0140	1.3931	0.0037	0.0084	0.0081
2.	C	1.3917	0.0000	1.4549	0.0140	0.1028	0.0120	0.9254	0.0036	0.0006
3.	C	0.0134	1.4549	0.0000	1.3647	0.0145	0.1055	0.0035	0.9260	0.0098
4.	C	0.0964	0.0140	1.3647	0.0000	1.3623	0.0124	0.0087	0.0033	0.0031
5.	C	0.0140	0.1028	0.0145	1.3623	0.0000	1.4515	0.0005	0.0101	0.9162
6.	C	1.3931	0.0120	0.1055	0.0124	1.4515	0.0000	0.0104	0.0006	0.0039
7.	H	0.0037	0.9254	0.0035	0.0087	0.0005	0.0104	0.0000	0.0034	0.0005
8.	H	0.0084	0.0036	0.9260	0.0033	0.0101	0.0006	0.0034	0.0000	0.0005
9.	H	0.0081	0.0006	0.0098	0.0031	0.9162	0.0039	0.0005	0.0005	0.0000
10.	H	0.0036	0.0106	0.0006	0.0090	0.0039	0.9245	0.0005	0.0006	0.0034
11.	C	0.0027	0.0097	0.0146	1.0776	0.0128	0.0096	0.0003	0.0018	0.0016
12.	C	0.0055	0.0004	0.0111	0.0025	0.0075	0.0004	0.0001	0.0001	0.0003
13.	C	0.0001	0.0002	0.0002	0.0013	0.0004	0.0001	0.0000	0.0000	0.0000

14.	N	0.0095	0.0008	0.0190	0.0133	0.0167	0.0009	0.0003	0.0003	0.0002
15.	O	0.0061	0.0007	0.0098	0.0138	0.0091	0.0005	0.0002	0.0003	0.0003
16.	C	0.0002	0.0002	0.0005	0.0010	0.0009	0.0003	0.0000	0.0000	0.0001
17.	O	0.0006	0.0004	0.0006	0.0014	0.0052	0.0010	0.0000	0.0001	0.0018
18.	N	0.0002	0.0002	0.0006	0.0017	0.0006	0.0002	0.0000	0.0001	0.0004
19.	C	0.0005	0.0005	0.0011	0.0014	0.0019	0.0009	0.0000	0.0000	0.0004
20.	H	0.0000	0.0000	0.0000	0.0000	0.0000	0.0000	0.0000	0.0000	0.0000
21.	H	0.0000	0.0000	0.0000	0.0000	0.0000	0.0000	0.0000	0.0000	0.0000
22.	H	0.0003	0.0002	0.0004	0.0005	0.0006	0.0001	0.0000	0.0000	0.0001
23.	H	0.0001	0.0001	0.0002	0.0007	0.0016	0.0002	0.0000	0.0000	0.0000
24.	H	0.0004	0.0000	0.0005	0.0004	0.0007	0.0000	0.0000	0.0000	0.0001
25.	H	0.0006	0.0000	0.0010	0.0000	0.0008	0.0000	0.0000	0.0000	0.0000
26.	C	1.0355	0.0107	0.0092	0.0016	0.0093	0.0108	0.0026	0.0004	0.0004
27.	H	0.0027	0.0112	0.0001	0.0055	0.0001	0.0108	0.0001	0.0000	0.0000
28.	H	0.0021	0.0027	0.0004	0.0011	0.0003	0.0093	0.0003	0.0000	0.0003
29.	H	0.0022	0.0097	0.0003	0.0015	0.0004	0.0031	0.0003	0.0002	0.0000
30.	H	0.0013	0.0003	0.0024	0.0028	0.0092	0.0002	0.0000	0.0003	0.0002

Atom		10	11	12	13	14	15	16	17	18
1.	C	0.0036	0.0027	0.0055	0.0001	0.0095	0.0061	0.0002	0.0006	0.0002
2.	C	0.0106	0.0097	0.0004	0.0002	0.0008	0.0007	0.0002	0.0004	0.0002
3.	C	0.0006	0.0146	0.0111	0.0002	0.0190	0.0098	0.0005	0.0006	0.0006
4.	C	0.0090	1.0776	0.0025	0.0013	0.0133	0.0138	0.0010	0.0014	0.0017
5.	C	0.0039	0.0128	0.0075	0.0004	0.0167	0.0091	0.0009	0.0052	0.0006
6.	C	0.9245	0.0096	0.0004	0.0001	0.0009	0.0005	0.0003	0.0010	0.0002
7.	H	0.0005	0.0003	0.0001	0.0000	0.0003	0.0002	0.0000	0.0000	0.0000
8.	H	0.0006	0.0018	0.0001	0.0000	0.0003	0.0003	0.0000	0.0001	0.0001

9.	H	0.0034	0.0016	0.0003	0.0000	0.0002	0.0003	0.0001	0.0018	0.0004
10.	H	0.0000	0.0003	0.0000	0.0000	0.0001	0.0001	0.0000	0.0001	0.0000
11.	C	0.0003	0.0000	0.2550	0.0056	1.3735	0.1666	0.0070	0.0160	0.0082
12.	C	0.0000	0.2550	0.0000	2.3825	0.0453	0.0511	1.0774	0.0829	0.0192
13.	C	0.0000	0.0056	2.3825	0.0000	0.0533	0.4364	0.0306	0.0586	0.0232
14.	N	0.0001	1.3735	0.0453	0.0533	0.0000	1.1459	0.0062	0.0076	0.0031
15.	O	0.0001	0.1666	0.0511	0.4364	1.1459	0.0000	0.0161	0.0078	0.0041
16.	C	0.0000	0.0070	1.0774	0.0306	0.0062	0.0161	0.0000	1.6166	1.1528
17.	O	0.0001	0.0160	0.0829	0.0586	0.0076	0.0078	1.6166	0.0000	0.1597
18.	N	0.0000	0.0082	0.0192	0.0232	0.0031	0.0041	1.1528	0.1597	0.0000
19.	C	0.0000	0.0191	0.0019	0.0016	0.9363	0.0404	0.0004	0.0005	0.0001
20.	H	0.0000	0.0002	0.0010	0.0007	0.0000	0.0001	0.0029	0.0149	0.8336
21.	H	0.0000	0.0000	0.0118	0.0026	0.0001	0.0005	0.0021	0.0040	0.8288
22.	H	0.0000	0.0091	0.0006	0.0008	0.0029	0.0026	0.0001	0.0001	0.0001
23.	H	0.0000	0.0009	0.0001	0.0006	0.0027	0.0123	0.0000	0.0001	0.0000
24.	H	0.0000	0.0119	0.0035	0.0037	0.0047	0.0026	0.0003	0.0005	0.0002
25.	H	0.0000	0.0151	0.0119	0.9111	0.0022	0.0097	0.0031	0.0011	0.0004
26.	C	0.0026	0.0006	0.0001	0.0000	0.0002	0.0001	0.0000	0.0000	0.0000
27.	H	0.0001	0.0002	0.0004	0.0000	0.0007	0.0004	0.0000	0.0000	0.0000
28.	H	0.0003	0.0000	0.0001	0.0000	0.0002	0.0001	0.0000	0.0000	0.0000
29.	H	0.0003	0.0000	0.0001	0.0000	0.0001	0.0001	0.0000	0.0000	0.0000
30.	H	0.0003	0.9080	0.0017	0.0029	0.0029	0.0041	0.0007	0.0007	0.0005

Atom		19	20	21	22	23	24	25	26	27
1.	C	0.0005	0.0000	0.0000	0.0003	0.0001	0.0004	0.0006	1.0355	0.0027
2.	C	0.0005	0.0000	0.0000	0.0002	0.0001	0.0000	0.0000	0.0107	0.0112
3.	C	0.0011	0.0000	0.0000	0.0004	0.0002	0.0005	0.0010	0.0092	0.0001

4.	C	0.0014	0.0000	0.0000	0.0005	0.0007	0.0004	0.0000	0.0016	0.0055
5.	C	0.0019	0.0000	0.0000	0.0006	0.0016	0.0007	0.0008	0.0093	0.0001
6.	C	0.0009	0.0000	0.0000	0.0001	0.0002	0.0000	0.0000	0.0108	0.0108
7.	H	0.0000	0.0000	0.0000	0.0000	0.0000	0.0000	0.0000	0.0026	0.0001
8.	H	0.0000	0.0000	0.0000	0.0000	0.0000	0.0000	0.0000	0.0004	0.0000
9.	H	0.0004	0.0000	0.0000	0.0001	0.0000	0.0001	0.0000	0.0004	0.0000
10.	H	0.0000	0.0000	0.0000	0.0000	0.0000	0.0000	0.0000	0.0026	0.0001
11.	C	0.0191	0.0002	0.0000	0.0091	0.0009	0.0119	0.0151	0.0006	0.0002
12.	C	0.0019	0.0010	0.0118	0.0006	0.0001	0.0035	0.0119	0.0001	0.0004
13.	C	0.0016	0.0007	0.0026	0.0008	0.0006	0.0037	0.9111	0.0000	0.0000
14.	N	0.9363	0.0000	0.0001	0.0029	0.0027	0.0047	0.0022	0.0002	0.0007
15.	O	0.0404	0.0001	0.0005	0.0026	0.0123	0.0026	0.0097	0.0001	0.0004
16.	C	0.0004	0.0029	0.0021	0.0001	0.0000	0.0003	0.0031	0.0000	0.0000
17.	O	0.0005	0.0149	0.0040	0.0001	0.0001	0.0005	0.0011	0.0000	0.0000
18.	N	0.0001	0.8336	0.8288	0.0001	0.0000	0.0002	0.0004	0.0000	0.0000
19.	C	0.0000	0.0000	0.0000	0.9352	0.9366	0.9279	0.0001	0.0000	0.0000
20.	H	0.0000	0.0000	0.0002	0.0000	0.0000	0.0000	0.0000	0.0000	0.0000
21.	H	0.0000	0.0002	0.0000	0.0000	0.0000	0.0000	0.0001	0.0000	0.0000
22.	H	0.9352	0.0000	0.0000	0.0000	0.0007	0.0005	0.0000	0.0000	0.0000
23.	H	0.9366	0.0000	0.0000	0.0007	0.0000	0.0005	0.0000	0.0000	0.0000
24.	H	0.9279	0.0000	0.0000	0.0005	0.0005	0.0000	0.0001	0.0000	0.0000
25.	H	0.0001	0.0000	0.0001	0.0000	0.0000	0.0001	0.0000	0.0000	0.0000
26.	C	0.0000	0.0000	0.0000	0.0000	0.0000	0.0000	0.0000	0.0000	0.9220
27.	H	0.0000	0.0000	0.0000	0.0000	0.0000	0.0000	0.0000	0.9220	0.0000
28.	H	0.0000	0.0000	0.0000	0.0000	0.0000	0.0000	0.0000	0.9401	0.0007
29.	H	0.0000	0.0000	0.0000	0.0000	0.0000	0.0000	0.0000	0.9379	0.0007
30.	H	0.0138	0.0000	0.0000	0.0002	0.0003	0.0001	0.0000	0.0000	0.0001

Atom		28	29	30
1.	C	0.0021	0.0022	0.0013
2.	C	0.0027	0.0097	0.0003
3.	C	0.0004	0.0003	0.0024
4.	C	0.0011	0.0015	0.0028
5.	C	0.0003	0.0004	0.0092
6.	C	0.0093	0.0031	0.0002
7.	H	0.0003	0.0003	0.0000
8.	H	0.0000	0.0002	0.0003
9.	H	0.0003	0.0000	0.0002
10.	H	0.0003	0.0003	0.0003
11.	C	0.0000	0.0000	0.9080
12.	C	0.0001	0.0001	0.0017
13.	C	0.0000	0.0000	0.0029
14.	N	0.0002	0.0001	0.0029
15.	O	0.0001	0.0001	0.0041
16.	C	0.0000	0.0000	0.0007
17.	O	0.0000	0.0000	0.0007
18.	N	0.0000	0.0000	0.0005
19.	C	0.0000	0.0000	0.0138
20.	H	0.0000	0.0000	0.0000
21.	H	0.0000	0.0000	0.0000
22.	H	0.0000	0.0000	0.0002
23.	H	0.0000	0.0000	0.0003
24.	H	0.0000	0.0000	0.0001
25.	H	0.0000	0.0000	0.0000
26.	C	0.9401	0.9379	0.0000

27. H 0.0007 0.0007 0.0001

28. H 0.0000 0.0006 0.0000

29. H 0.0006 0.0000 0.0000

30. H 0.0000 0.0000 0.0000
